# Colchicine for COVID-19 in the community (PRINCIPLE): a randomised, controlled, adaptive platform trial

**DOI:** 10.3399/BJGP.2022.0083

**Published:** 2022-04-20

**Authors:** Jienchi Dorward, Ly-Mee Yu, Gail Hayward, Benjamin R Saville, Oghenekome Gbinigie, Oliver Van Hecke, Emma Ogburn, Philip H Evans, Nicholas PB Thomas, Mahendra G Patel, Duncan Richards, Nicholas Berry, Michelle A Detry, Christina Saunders, Mark Fitzgerald, Victoria Harris, Milensu Shanyinde, Simon de Lusignan, Monique I Andersson, Christopher C Butler, FD Richard Hobbs, the PRINCIPLE Trial Collaborative Group

**Affiliations:** Nuffield Department of Primary Care Health Sciences, University of Oxford, Oxford, UK; Centre for the AIDS Programme of Research in South Africa (CAPRISA), University of KwaZulu–Natal, Durban, South Africa.; Nuffield Department of Primary Care Health Sciences, University of Oxford, Oxford, UK.; Nuffield Department of Primary Care Health Sciences, University of Oxford, Oxford, UK.; Berry Consultants, Texas, TX, US; Department of Biostatistics, Vanderbilt University School of Medicine, Tennessee, TN, US.; Nuffield Department of Primary Care Health Sciences, University of Oxford, Oxford, UK.; Nuffield Department of Primary Care Health Sciences, University of Oxford, Oxford, UK.; Nuffield Department of Primary Care Health Sciences, University of Oxford, Oxford, UK.; College of Medicine and Health, University of Exeter, Exeter, UK; National Institute for Health Research (NIHR) Clinical Research Network, NIHR, London, UK.; NIHR, London; Royal College of General Practitioners, London, UK.; Nuffield Department of Primary Care Health Sciences, University of Oxford, Oxford, UK.; Oxford Clinical Trials Research Unit, Botnar Research Centre, University of Oxford, Oxford, UK.; Berry Consultants, Texas, TX, US.; Berry Consultants, Texas, TX, US.; Berry Consultants, Texas, TX, US.; Berry Consultants, Texas, TX, US.; Nuffield Department of Primary Care Health Sciences, University of Oxford, Oxford, UK.; Nuffield Department of Primary Care Health Sciences, University of Oxford, Oxford, UK.; Nuffield Department of Primary Care Health Sciences, University of Oxford, Oxford, UK; Royal College of General Practitioners, London, UK.; Nuffield Department of Clinical Medicine, University of Oxford, Oxford, UK.; Nuffield Department of Primary Care Health Sciences, University of Oxford, Oxford, UK.; Nuffield Department of Primary Care Health Sciences, University of Oxford, Oxford, UK.

**Keywords:** colchicine, community, COVID-19, primary health care, randomised controlled trial

## Abstract

**Background:**

Colchicine has been proposed as a COVID-19 treatment.

**Aim:**

To determine whether colchicine reduces time to recovery and COVID-19-related admissions to hospital and/or deaths among people in the community.

**Design and setting:**

Prospective, multicentre, open-label, multi-arm, randomised, controlled, adaptive platform trial (PRINCIPLE).

**Method:**

Adults aged ≥65 years or ≥18 years with comorbidities or shortness of breath, and unwell for ≤14 days with suspected COVID-19 in the community, were randomised to usual care, usual care plus colchicine (500 µg daily for 14 days), or usual care plus other interventions. The co-primary endpoints were time to first self-reported recovery and admission to hospital/death related to COVID-19, within 28 days, analysed using Bayesian models.

**Results:**

The trial opened on 2 April 2020. Randomisation to colchicine started on 4 March 2021 and stopped on 26 May 2021 because the prespecified time to recovery futility criterion was met. The primary analysis model included 2755 participants who were SARS-CoV-2 positive, randomised to colchicine (*n* = 156), usual care (*n* = 1145), and other treatments (*n* = 1454). Time to first self-reported recovery was similar in the colchicine group compared with usual care with an estimated hazard ratio of 0.92 (95% credible interval (CrI) = 0.72 to 1.16) and an estimated increase of 1.4 days in median time to self-reported recovery for colchicine versus usual care. The probability of meaningful benefit in time to recovery was very low at 1.8%. COVID-19-related admissions to hospital/deaths were similar in the colchicine group versus usual care, with an estimated odds ratio of 0.76 (95% CrI = 0.28 to 1.89) and an estimated difference of −0.4% (95% CrI = −2.7 to 2.4).

**Conclusion:**

Colchicine did not improve time to recovery in people at higher risk of complications with COVID-19 in the community.

## INTRODUCTION

Colchicine is widely used for the treatment and prophylaxis of gout. Colchicine inhibits cellular transport and mitosis by binding to tubulin and preventing its polymerisation as part of the cytoskeleton transport system.^1^ Although the precise mechanism is unclear, colchicine has an inhibitory action on the NLRP3 inflammasome.^1^ Inflammasomes are activated in COVID-19 and the degree of activation is correlated with disease severity.^2^ Colchicine is therefore an attractive candidate to test the role of the inflammasome in COVID-19.^3^

Several observational studies and one small randomised controlled trial suggested that colchicine may be an effective treatment for patients admitted to hospital with COVID-19.^4^^–^7 However, the large RECOVERY trial of patients with COVID-19 admitted to hospital clearly demonstrated that colchicine did not improve the primary outcome of 28-day mortality, or any secondary outcomes, when compared with usual care.^8^ Although the actions of colchicine may be more relevant earlier in the disease to prevent the progression from inflammatory activation to a hyperinflammatory state,^3^ evidence for effectiveness of colchicine in the community is lacking. The COLCORONA randomised controlled trial, among 4488 people aged ≥40 years with suspected COVID-19 in the community, was stopped early for administrative reasons and did not reach the pre-specified superiority criterion for a reduction in the primary outcome of COVID-19-related hospital admissions/death.^9^ However, in a pre-specified secondary analysis among participants who were polymerase chain reaction (PCR) positive for SARS-CoV-2, there was a marginally significant reduction in COVID-19-related admissions to hospital and deaths compared with placebo (4.6% versus 6.0%; odds ratio (OR) 0.75, 95% confidence interval [CI] = 0.57 to 0.99, *P* = 0.042).

**Table 1:** How this fits in

Colchicine has been proposed as a treatment for COVID-19 because of its anti-inflammatory properties, but evidence to support its use is inconclusive, and its effect on time to recovery in the community has not been evaluated. The RECOVERY trial found no benefit with colchicine use among people admitted to hospital with COVID-19, whereas the COLCORONA trial found some evidence of a 1.1% and 1.4% absolute reduction in admissions to hospital/deaths among adults with suspected or confirmed COVID-19 in the community, respectively. In this national, randomised, controlled, adaptive platform trial, evidence was found of no meaningful benefit with colchicine on time to recovery, and, because the threshold for futility on time to recovery was met, randomisation to colchicine was stopped before collecting substantial data on admissions to hospital and death, leading to imprecise estimates for that outcome. These findings add to the evidence currently available and suggest that colchicine should not be recommended for treating symptoms of COVID-19.

Pulmonary emboli and gastrointestinal adverse events were significantly higher in the colchicine arm, and the trial did not measure time to recovery. Although encouraging, these data are generally considered insufficient to support a recommendation and several key bodies have called for more information.^10^^–^12

This current study aimed to determine whether colchicine speeds recovery and reduces COVID-19-related hospital admissions or deaths in people in the community.

## METHOD

### Trial design

The effectiveness of colchicine was assessed in the UK national, multicentre, primary care, open-label, multi-arm, prospective adaptive Platform Randomised trial of Treatments in the Community for Pandemic and Epidemic Illnesses (PRINCIPLE), which opened on 2 April 2020, and is ongoing. Details of the PRINCIPLE Collaborative Group are listed in Supplementary Appendix S1, and the protocol is available in Supplementary Appendix S2 and also at the trial website, https://www.principletrial.org. A ‘platform trial’ allows multiple treatments for the same disease to be tested simultaneously. A master protocol defines prospective decision criteria for dropping interventions for futility, declaring interventions superior, or adding new interventions.^13^ This allows interventions with little evidence of meaningful benefit to be rapidly dropped for futility and replaced by new interventions, thereby directing resources towards identifying community-based treatments for COVID-19. Interventions evaluated in PRINCIPLE include hydroxychloroquine, azithromycin,^14^ doxycycline,^15^ inhaled budesonide,^16^ favipiravir, ivermectin, and, reported here, colchicine.

The UK Medicines and Healthcare products Regulatory Agency and the South Central-Berkshire Research Ethics Committee (reference: 20/SC/0158) approved the trial protocol. Online consent was obtained from all participants. The authors vouch for the accuracy and completeness of the data and for fidelity to the protocol. An independent Trial Steering Committee and Data Monitoring and Safety Committee provided trial oversight.

### Participants

From the beginning of the trial, people in the community were eligible if they were aged ≥65 years, or 50–65 years with comorbidities (see Supplementary Appendix S2, section 2.1.2 Inclusion criteria), and had ongoing symptoms from PCR-confirmed COVID-19 or suspected COVID-19 (in accordance with the UK NHS definition of high temperature and/or new, continuous cough and/or change in sense of smell/taste)^17^^,^^18^ that had started within the previous 14 days. When the colchicine arm opened, eligibility criteria were expanded to allow enrolment of people aged 18–65 years with comorbidities or shortness of breath.^19^ Comorbidities required for eligibility were: heart disease; hypertension; asthma or lung disease; diabetes; hepatic impairment; stroke or neurological problems; weakened immune system (for example, chemotherapy); and self-reported obesity or body mass index ≥35 kg/m^2^. People were ineligible to be randomised to colchicine if they were already taking colchicine or if colchicine was contraindicated according to the British National Formulary.

Initially, eligible people were recruited, screened, and enrolled through participating general medical practices, but from 17 May 2020 people across the UK could enrol online or by telephone. After patients completed a baseline and screening questionnaire, a clinician or trained research nurse confirmed eligibility using the patient’s primary care medical record, accessed remotely where necessary, before randomisation. Several community outreach strategies were implemented aiming to increase recruitment of those from ethnically diverse communities and socioeconomically deprived backgrounds, who have been disproportionally affected by COVID-19.^20^

### Randomisation and masking

Eligible, consenting participants were randomised using a secure, in-house, web-based randomisation system (Sortition version 2.3). Randomisation was stratified by age (<65 years/≥65 years) and presence of comorbidity (yes/no), and probabilities were determined using response adaptive randomisation via regular interim analyses, which allows allocation of more participants to interventions with better observed time to recovery outcomes (see Supplementary Appendix S3, section 13.7 Plans for analysis of colchicine). However, between 31 March 2021 and 8 April 2021, only the colchicine and usual care arms were active, with 1:1 allocation between each. The trial team was masked to randomisation probabilities.

### Trial procedures

Participants were followed up through an online, daily symptom diary for 28 days after randomisation, supplemented with telephone calls to non-responders on days 7, 14, and 28. The diary includes questions about illness recovery (ascertained by answering the question, ‘Do you feel recovered today? [that is symptoms associated with illness are no longer a problem] Yes/No’); overall illness severity (a rating of how well they are feeling on a scale of 1–10 [1 being the worst and 10 being the best]); individual symptom severity on a four-point scale (0, no problem to 3, major problem); and healthcare service utilisation. Participants could nominate a relative or carer as a trial partner to help provide follow-up data. Consent was obtained to ascertain healthcare use outcome data from general practice and hospital records. The aim was to provide a self-swab for SARSCoV-2 confirmatory PCR testing, but capacity issues early in the pandemic meant testing was unavailable for some participants.

### Trial interventions

Participants received usual care plus colchicine 500 µg daily for 14 days, or usual care alone. Colchicine was either prescribed or issued directly by the participant’s general medical practitioner, or issued centrally by the study team and delivered to the participant by urgent courier. Usual care in the UK NHS for suspected COVID-19 in the community is largely focused on managing symptoms with antipyretics,^21^ although previous results from PRINCIPLE^16^ led to the introduction of inhaled budesonide on an off-label, case-by-case basis for people aged ≥65 years or 50–65 years with comorbidities.^22^

### Primary outcomes

The trial commenced with the primary outcome of COVID-19-related hospital admission or death within 28 days. However, hospital admission rates in the UK^23^ were lower than initially expected.^24^ Therefore, the Trial Management Group and Trial Steering Committee recommended amending the primary outcome to also include illness duration,^25^^,^^26^ which is an important outcome for patients and has a substantial economic and social impact. This received ethical approval on 16 September 2020, and was implemented before performing any interim analyses. Thus, the trial has two co-primary endpoints measured within 28 days of randomisation:

- time to first-reported recovery defined as the first instance that a participant reports feeling recovered; and
- admission to hospital or death related to COVID-19.

Decisions about COVID-19 relatedness were made after independent review of available data by two clinicians masked to treatment allocation and study identifiers.

### Secondary outcomes

Secondary outcomes (see Supplementary Appendix S3, section 3.3 Secondary outcomes) include a binary outcome of early, sustained recovery (recovered by day 14 and remains recovered until day 28), time to sustained recovery (date participant first reports recovery and subsequently remains well until 28 days), daily rating from one to ten of how well participants feel, time to initial alleviation of symptoms (date symptoms first reported as minor or none), time to sustained alleviation of symptoms (date symptoms first reported as minor or none and subsequently remain minor or none until 28 days), time to initial reduction of severity of symptoms (among people with symptom at baseline, date symptom severity reported at least one grade lower), worsening of symptoms (worsening symptom by one grade from mild to moderate/severe, or from moderate to severe, and excluding individuals reporting symptom severity as major at baseline), contacts with healthcare services, hospital assessment without admission, duration of hospital admission, oxygen administration, intensive care unit admission, mechanical ventilation, World Health Organization (WHO) ordinal scale of clinical progression, adherence to study treatment, WHO-5 Well-Being Index,^27^ serious adverse events, all-cause death or urgent, non-elective hospital admission, and reports of new household infections.

All time to event analyses used date of randomisation as baseline. Secondary outcomes that capture sustained recovery were included because of the often recurrent and relapsing nature of COVID-19 symptoms.

### Statistical analysis

Sample size calculation and statistical analysis are detailed in Supplementary Appendix S4 (the Adaptive Design Report) and Supplementary Appendix S3 (the Master Statistical Analysis Plan). In the Adaptive Design Report (see Supplementary Appendix S4) the authors justify sample sizes by simulating the operating characteristics of the adaptive design in multiple scenarios, which explicitly account for response adaptive randomisation, early stopping for futility/success, and multiple interventions. In brief, for the primary outcome analyses, assuming a median time to recovery of 9 days in the usual care group, approximately 400 participants per group would provide 90% power to detect a 2-day difference in median recovery time. Assuming 5% hospital admissions in the usual care group, approximately 1500 participants per group would provide 90% power to detect a 50% reduction in the relative risk of admission to hospital/death.

The first co-primary outcome, time to first self-reported recovery, was analysed using a Bayesian piecewise exponential model. The second co-primary outcome, admission to hospital/death, was analysed using a Bayesian logistic regression model. Both models were regressed on treatment group and stratification covariates (age <65 years/≥65 years and comorbidity yes/no). These primary outcomes were evaluated using a ‘gate-keeping’ strategy to preserve the overall type I error without additional adjustments for multiple hypotheses.

The hypothesis for the time to first recovery endpoint was evaluated first, and, if the null hypothesis was rejected, the hypothesis for the second co-primary endpoint of admission to hospital/death was evaluated. In the context of multiple interim analyses, the master protocol specifies that each null hypothesis is rejected if the Bayesian posterior probability of superiority exceeded 0.99 for the time to recovery endpoint and 0.975 (via gate-keeping) for the admission to hospital/death endpoint. For the purposes of defining futility rules, a clinically meaningful hazard ratio (HR) for time to first-reported recovery of ≥1.2 (equating to approximately 1.5 days’ difference in median time to recovery, assuming 9 days’ recovery in the usual care arm) was pre-specified, and a clinically meaningful OR of ≤0.80 for admissions to hospital/deaths (equating to approximately a 1% decrease in the hospital admission rate, assuming a rate of 5% in the usual care arm) was pre-specified. If there is insufficient evidence of a clinically meaningful benefit in time to recovery, futility is declared and randomisation to that intervention is stopped, meaning other interventions can be evaluated more rapidly in the trial. For each primary outcome endpoint (time to recovery and admission to hospital/death), a model-based estimate of absolute benefit (days and per cent, respectively) was obtained by applying the model-based estimate of treatment benefit (HR or OR, respectively) to a bootstrap sample of the concurrent and eligible usual care population.

At the beginning of the trial, because of initial difficulties with community SARSCoV-2 PCR testing in the UK, participants with suspected COVID-19 were included in the primary analysis population, irrespective of confirmatory testing. When testing became more accessible, the Trial Steering Committee recommended restricting the primary analysis population to those with confirmed COVID-19. This change was included in protocol version 7.1 on 22 February 2021 and approved on 15 March 2021, before any interim colchicine results were disclosed to the Trial Management Group. Therefore, the pre-specified primary analysis population includes all eligible participants positive for SARS-CoV-2 randomised to colchicine, usual care, and other interventions, from the start of the platform trial until the colchicine arm was closed, on 26 May 2021. This population includes participants randomised to usual care before the colchicine group opened, who may differ from concurrently randomised participants because of changes in the inclusion/exclusion criteria (for example, participants aged ≥18 years with comorbidity or shortness of breath became eligible when the colchicine group opened), and changes over time in the predominant variant and amount of circulating SARS-CoV-2 or usual care, including increasing availability of vaccinations. Therefore, the primary analysis models include parameters to adjust for potential temporal drift in the trial population, by estimating the primary endpoint in the usual care group across time via Bayesian hierarchical modelling.

A key pre-specified sensitivity analysis of the primary outcomes was also conducted using the concurrent randomised population; this was defined as all participants who were positive for SARS-CoV-2 randomised during the time period when the colchicine arm was active. To determine the applicability of the results to situations where PCR testing may not be readily available, secondary analyses of time to recovery and COVID-19-related admission to hospital/death among the overall study population were conducted, irrespective of SARS-CoV-2 status.

Analyses of all secondary outcomes, and pre-specified subgroup analyses, were conducted using participants who were eligible for colchicine and positive for SARSCoV-2 and concurrently randomised to colchicine or usual care: the concurrently randomised and eligible SARS-CoV-2 positive population. Secondary time to event outcomes were analysed using Cox proportional hazard models, and binary outcomes were analysed using logistic regression, adjusting for comorbidity, age, duration of illness, and vaccination status. As a result of the high proportion contributing to the analysis of primary outcomes (95%), the potential impact of missing data was not explored. All model assumptions were evaluated. Analyses were conducted using R (version 4.0.3) and Stata (version 16.1).

### Patient and public involvement

A patient and public involvement group was convened of five women and two men, who provided input into the patient-facing materials, choice of trial outcomes, and delivery plans. They were very supportive of the option of a trial partner to assist participants, and of the work to evaluate community treatments for COVID-19. Two public contributors serve on the Trial Steering Committee and continue to provide input on patient-facing material, trial design, and dissemination.

Feedback from a UK-wide survey of 291 PRINCIPLE participants showed that 90% found the study information prepared them for participation and 94% felt they were treated well by the study team. Improvements were made in communications about study procedures following this detailed feedback. Patient and public involvement support has been forthcoming from a diverse range of ethnic minority communities for widespread promotion of the study, for example, in community and faith meetings, and broadcast and print media.^20^

## RESULTS

### Population

The first participant was randomised into PRINCIPLE on 2 April 2020. Enrolment into the colchicine group started on 4 March 2021. On 26 May 2021, the Trial Steering Committee advised the Trial Management Group to stop randomisation to colchicine because the prespecified futility criterion had been met on time to recovery. Those participants taking colchicine at the time randomisation was stopped were advised that evidence for futility had been identified. All participants were followed up for the full 28 days.

**Figure 1.**
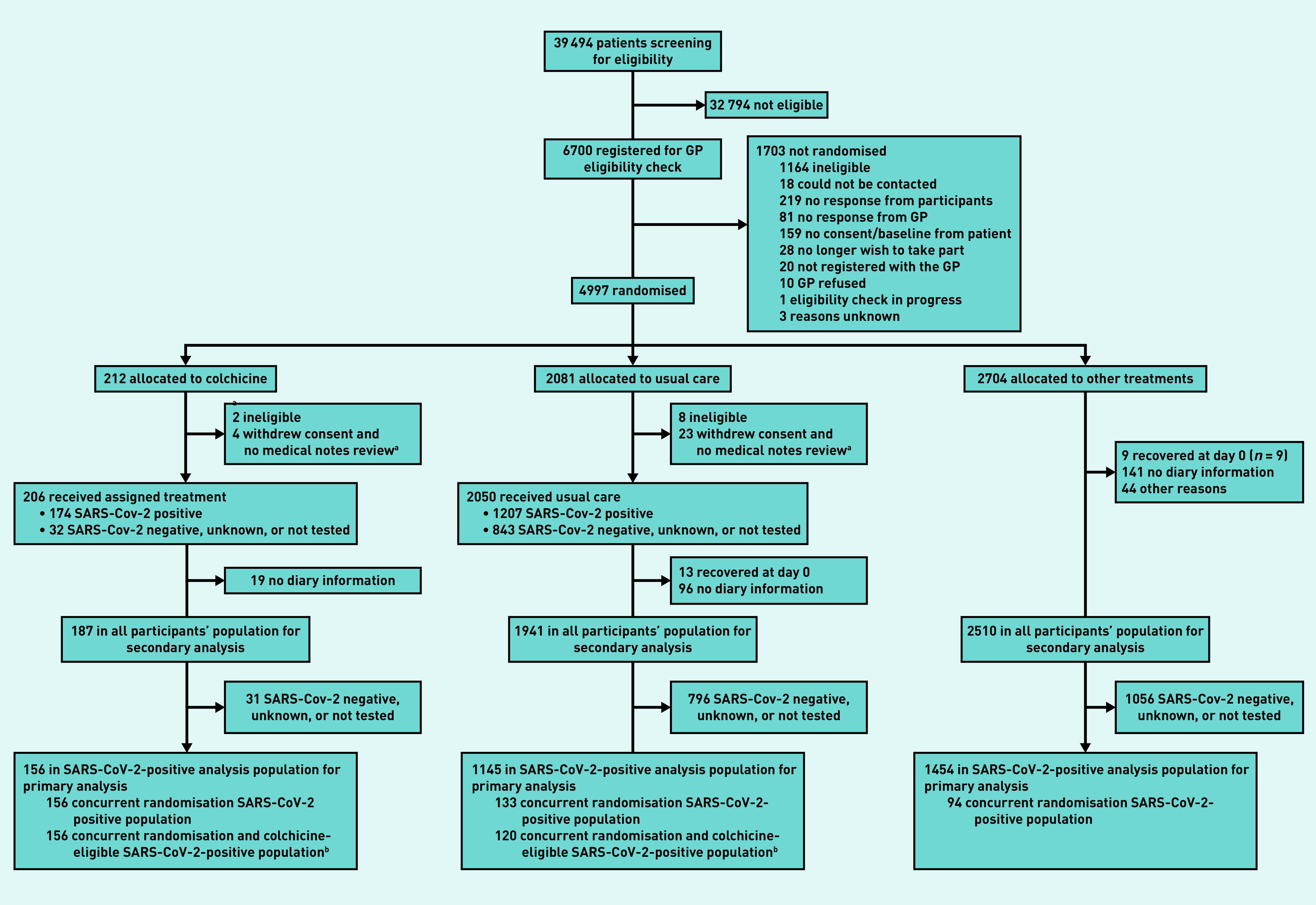
*Participant flow diagram.* *^a^****Participants provided no diary information.*** *^b^****Analysis for secondary outcomes.***

In total, 4997 participants were randomised of whom 212 were allocated to colchicine, 2081 to usual care alone, and 2704 to other treatments (Figure 1); 4221 of 4880 (86.5%) eligible participants had a SARS-CoV-2 test result available, of which 2900 (68.7%) tested positive.

The Bayesian primary analysis model includes data from 2755 of 2900 (95.0%) participants who were SARS-CoV-2 positive and who provided follow-up data and were randomised to colchicine (*n* = 156), usual care alone (*n* = 1145), and other treatment groups (*n* = 1454). To protect the integrity of the platform trial and other interventions, only descriptive summaries of participants randomised to colchicine and usual care are provided in this article.

The average age (range) of participants was 61 years (18–100 years), 1260 (91.2%) were of white ethnicity, and 1165 (84.4%) had comorbidities (percentages calculated from primary analysis population data in Supplementary Table S1, *N* = 1381). At randomisation, median time from symptom onset was 6 days (interquartile range 4–9 days). Baseline characteristics were similar between the comparison groups (see Supplementary Tables S1 and S2). Data regarding inhaled corticosteroid was not consistently recorded early in the trial, but, in the concurrent randomisation analysis population, 13/174 (7%) of the colchicine arm and 14/140 (10%) of the usual care arm reported taking inhaled corticosteroids at randomisation or during follow-up.

Of 184 participants randomised to colchicine who provided medication use information, 138 (75%) reported taking colchicine for at least 7 days.

### Primary outcomes

**Figure 2.**
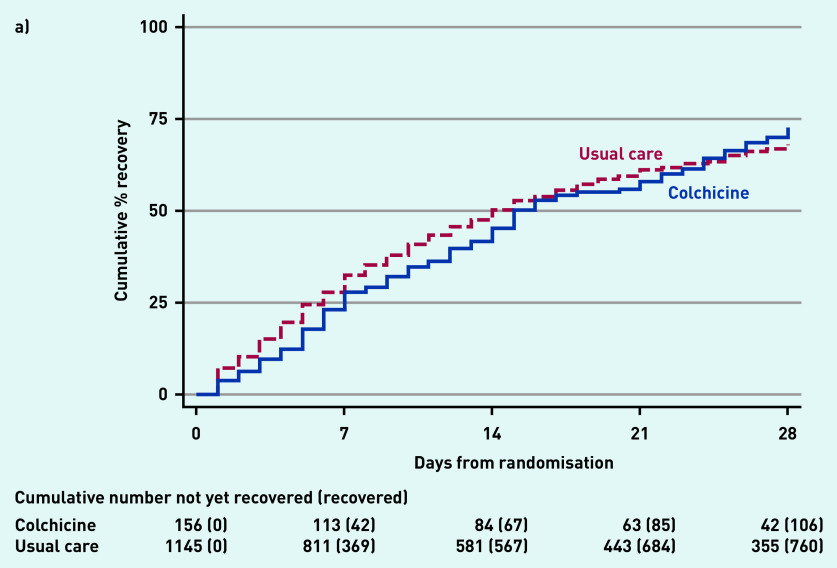

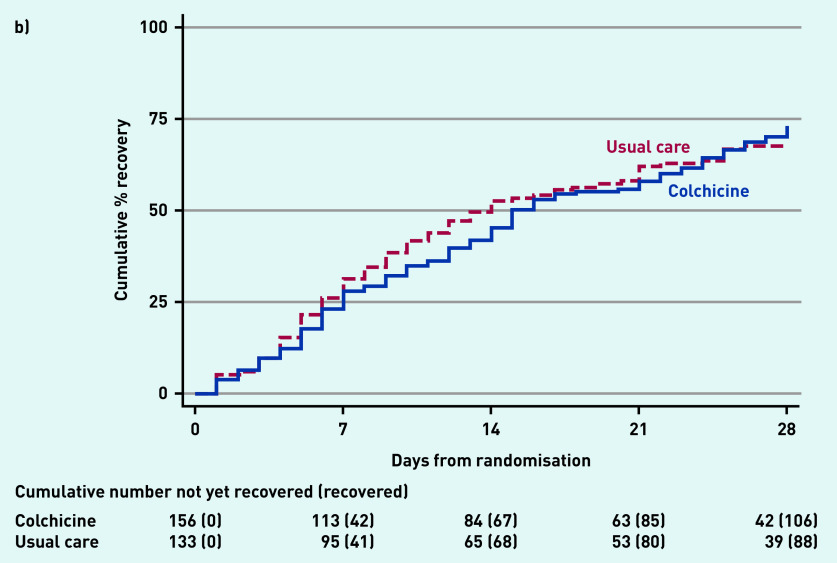
*Time to first-reported recovery. a) SARS-CoV-2-positive primary analysis population. b) Concurrent randomisation SARS-CoV-2-positive population.*

In the SARS-CoV-2-positive primary analysis population, the observed median time to first recovery was 15 days in the colchicine group compared with 14 days in the usual care group (Figure 2a). In the concurrent randomisation analysis population (excluding participants randomised to usual care before the colchicine arm opened) the observed median time to first recovery was 15 days in the colchicine group and 14 days in the usual care group.

Based on the Bayesian primary analysis model, which adjusts for temporal drift, there was no evidence of a benefit in time to first recovery in the colchicine group versus usual care (HR 0.92, 95% Bayesian credible interval [CrI] = 0.72 to 1.16). Based on a bootstrap estimated median time to recovery of 13 days in the concurrent and eligible usual care SARS-CoV-2-positive population, the model-based estimated HR corresponds to an estimated 1.14 (95% CrI = −1.86 to 5.21) additional days in median time to first-reported recovery for colchicine relative to usual care. The probability that time to recovery was shorter in the colchicine group versus usual care (that is probability of superiority) was 0.241, which did not meet the pre-specified superiority threshold of 0.99. The probability of meaningful effect (pre-specified as an HR ≥1.2 for the purpose of evaluating futility) was 0.018 (see Supplementary Table S3). The low probability of a clinically meaningful treatment effect was consistent in the concurrent randomisation and overall study population.

In the SARS-CoV-2-positive primary analysis population, there were 6/156 (3.8%) COVID-19-related admissions to hospital/deaths in the colchicine group (six admissions to hospital, no deaths), and 119/1145 (10.4%) in the usual care group (116 admissions to hospital, of whom nine died, and three deaths without hospital admission). The high levels of admissions to hospital/deaths in the usual care group in the primary analysis population were driven by the high event rate before the colchicine arm opened. In the concurrent randomisation analysis population, which excluded participants randomised to usual care before the colchicine arm opened, there were 4/133 (3.0%) COVID-19-related admissions to hospital/deaths in the usual care group (three admissions to hospital, and one death without hospital admission).

In the Bayesian primary analysis model, which takes into account the temporal change in event rates, COVID-19-related admission to hospital/deaths in the colchicine group compared with usual care were similar, with an estimated OR of 0.76 (95% CrI = 0.28 to 1.89). Based on a bootstrap estimated hospital admission rate of 2.5% in the concurrent and eligible usual care population, the model-based estimated OR corresponds to an estimated difference in the hospital admission rate of −0.4% (95% CrI = −2.7% to 2.4) (see Supplementary Table S3). The probability that COVID-19-related admissions to hospital/deaths were lower in the colchicine arm versus usual care (that is probability of superiority) was 0.714 and, because superiority was not reached on time to recovery, the admission to hospital/death outcome was not formally analysed for significance because of the gate-keeping hypothesis structure. The probability that there was a meaningful reduction in COVID-19-related admissions to hospital/deaths (predefined as an OR of ≤0.80) was 0.585.

The point estimates of the model-based effects on admissions to hospital were not consistent across the primary analysis population (OR 0.76, 95% CrI = 0.28 to 1.89), the concurrent randomisation population (OR 1.31, 95% CrI = 0.41 to 4.21), and the overall study population (OR 0.72, 95% CrI = 0.27 to 1.77). This is because of the very small number of events observed in the concurrent analysis population and corresponds to wide credible intervals.

The general conclusion is consistent across these populations, in that there is lack of evidence of a clinically meaningful treatment effect on admissions to hospital/deaths (see Supplementary Table S3).

### Secondary outcomes

Analyses of secondary outcomes, using the concurrent randomisation and eligible SARS-CoV-2-positive population, are presented in Supplementary Table S3 and Supplementary Figures S1–S3. There was no clear evidence of benefit for any of the secondary outcomes.

**Figure 3.**
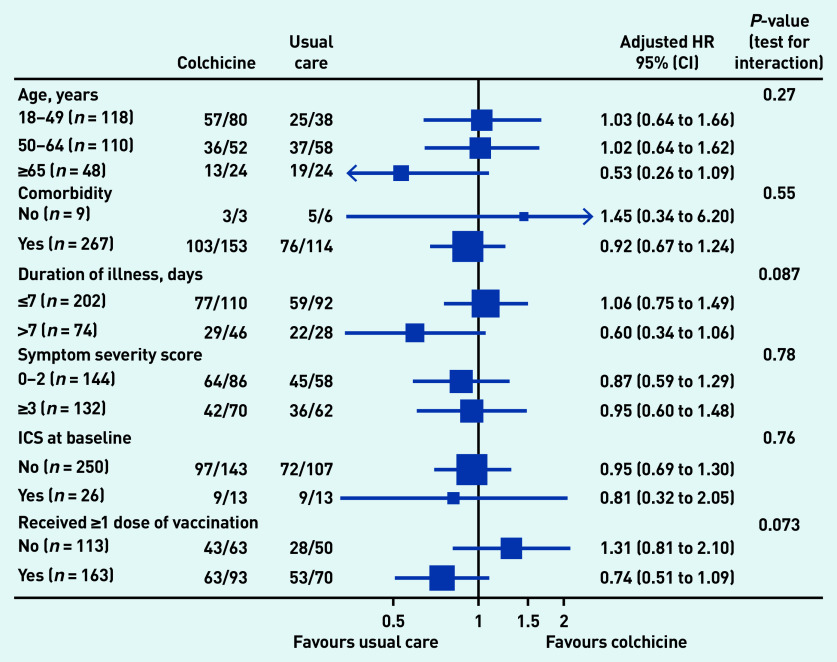
*Forest plot of subgroup analysis of time to first-reported recovery (concurrent randomisation and eligible SARS-CoV-2-positive population. HR = hazard ratio. ICS = inhaled corticosteroids.*

In the pre-specified subgroup analyses, there was no strong statistical evidence that symptom duration before randomisation, baseline illness severity score, inhaled corticosteroid use, age, or comorbidity modified the effect of colchicine on time to first-reported recovery (Figure 3), although numbers were small. In post hoc subgroup analyses, there was no evidence that colchicine effects differed by vaccination status (Figure 3), although numbers were small. Event rates were too low to conduct subgroup analyses of the admission to hospital/death outcome. Regarding serious adverse events, there was one hospital admission unrelated to COVID-19 in the colchicine group and one in usual care (see Supplementary Table S4).

## DISCUSSION

### Summary

This analysis from a platform, randomised trial involving people in the community with COVID-19 found that colchicine did not meaningfully improve time to first recovery compared with usual care alone. There was also no evidence of a difference in secondary measures of wellbeing, sustained recovery, symptoms, or healthcare service use. In line with the protocol, because of futility reaching the futility criterion on time to recovery, the colchicine arm was stopped before collecting substantial data on admissions to hospital/deaths, meaning the estimates of effect on hospital admissions had wide credible intervals. Overall, these findings do not support the use of colchicine 500 µg daily for 14 days as treatment for the symptoms of COVID-19 in the community.

### Strengths and limitations

Strengths include the pragmatic design of the PRINCIPLE trial, which allowed for efficient evaluation of the effectiveness of colchicine as an early, standalone intervention, as it might be used in the community. The focus was on patients at increased risk of complications and routine electronic health records were used to confirm admission to hospital/death; primary outcome data on >95% of participants were obtained. Although the primary analysis was restricted to patients who were SARS-CoV-2 positive, the secondary analyses of the co-primary outcomes was conducted among patients with suspected COVID-19 but without PCR-confirmed SARS-CoV-2 infection, as limited SARS-CoV-2 testing may necessitate early empirical treatment in low-resource settings. Furthermore, variation in PCR testing sensitivity, particularly if self-administered, means some participants will have had false-negative tests.^28^ Time to recovery estimates were similar in the SARSCoV-2-positive population, all participants irrespective of SARS-CoV-2 status, as well as the concurrent randomisation SARSCoV-2-positive population (the latter populations are most analogous to those in traditional two arm trials).

Although the sample size of the colchicine group in PRINCIPLE was relatively small, the Bayesian primary analysis model leverages previous enrolments in the usual care arm to increase the precision of estimates, which allow the authors to declare futility with high precision because of a very low probability of a meaningful benefit of colchicine on time to recovery. This allowed the colchicine arm to be dropped and for trial resources to be rapidly redirected to other interventions, with the ultimate aim of identifying effective treatments for COVID-19 in the community, attesting to the efficiency of the trial design in keeping with the aim of rapid generation of evidence for use within the pandemic itself.

A pragmatic, open-label design was used, similar to other large COVID-19 platform trials,^29^^,^^30^ to evaluate the addition of colchicine to usual care, rather than to assess the benefit of colchicine compared with a placebo. Time to recovery was a self-reported outcome, which could have been influenced by participants being aware of treatment allocation. However, if a positive placebo effect influenced the self-reported time to recovery outcome, it would likely be masking an even greater negative effect of colchicine. This outcome was used as it was of greatest interest to the patient and public contributors, and is best ascertained by direct patient report, rather than by the use of surrogate measures. It was hypothesised that a treatment that does not reduce recovery time is also unlikely to reduce COVID-19-related admissions to hospital/death. However, it is possible for a treatment to reduce the likelihood of severe disease without reducing duration of the illness, or for a treatment to cause side effects that delay recovery.

### Comparison with existing literature

To the authors’ knowledge, PRINCIPLE is the first randomised trial to evaluate the effect of colchicine on time to recovery for people with COVID-19 in the community. The study finding of no benefit contrasts with some of the findings of the COLCORONA trial, which was terminated early at approximately 75% of targeted recruitment and which found a possible reduction in admissions to hospital/deaths with colchicine, although time to recovery was not measured.^9^ The primary endpoint of COVID-19-related hospital admission or death occurred in 104/2235 (4.7%) in the colchicine group and 131/2253 (5.8%) in the placebo group (OR 0.79, 95.1% CI = 0.61 to 1.03, *P* = 0.08).^9^ In the 4159 patients who were SARSCoV-2 positive, the primary endpoint was lower in the colchicine arm compared with placebo (4.6% versus 6.0%, OR 0.75, 95% CI = 0.57 to 0.99, *P* = 0.04).^9^ Diarrhoea (13.7% versus 7.3%), gastrointestinal adverse events (23.9% versus 14.8%), and pulmonary emboli (0.5% versus 0.1%) were higher in the colchicine group compared with placebo.^9^ Participants in the COLCORONA trial were slightly older than PRINCIPLE (median age in colchicine arm 53 years [COLCORONA] versus 48 years [PRINCIPLE]). In PRINCIPLE, taking into account the pharmacokinetic variability and narrow therapeutic index of colchicine, a moderate dose was selected with no loading dose. The loading dose used in the COLCORONA study may mean intracellular concentrations rose faster and may explain the difference in clinical effect, including the higher proportion of gastrointestinal adverse events and diarrhoea seen in the COLCORONA colchicine arm. The current study did not find evidence that colchicine led to a worsening of any symptoms, although there was a trend towards a longer duration of diarrhoea symptoms, and this could have played a role in prolonging time to self-reported recovery. In the RECOVERY trial among 11 340 people admitted to hospital with COVID-19, there was no evidence that colchicine improved the primary outcome of 28-day mortality.^8^

### Implications for research and practice

Although the COLCORONA trial found limited evidence that colchicine may reduce COVID-19-related admission to hospital/death,^9^ most treatment guidelines have not recommended colchicine’s use in the community, potentially because of its increased gastrointestinal side effect profile and the observed higher rates of pulmonary embolism. The results of the current study support this position; no evidence was found in this study that colchicine at a dose of 500 µg once daily shortens time to recovery among patients with COVID-19 in the community. Therefore, with the evidence available to date, colchicine should neither be used for treatment of COVID-19 in community nor hospital settings. The authors of the current study are planning analyses of the effect of colchicine on longer-term symptoms related to ‘long COVID’.

In conclusion, colchicine does not improve time to recovery for adults with COVID-19 in the community, and there was no evidence for a benefit in COVID-19-related admissions to hospital/deaths but estimates were imprecise because of the few admissions to hospital.

## Data

Data can be shared with qualifying researchers who submit a proposal with a valuable research question as assessed by a committee formed from the Trial Management Group including senior statistical and clinical representation. A contract should be signed.
